# Identification of ZYG11A as a candidate IGF1-dependent proto-oncogene in endometrial cancer

**DOI:** 10.18632/oncotarget.27055

**Published:** 2019-07-09

**Authors:** Laris Achlaug, Rive Sarfstein, Karthik Nagaraj, Lena Lapkina-Gendler, Ilan Bruchim, Manisha Dixit, Zvi Laron, Shoshana Yakar, Haim Werner

**Affiliations:** ^1^ Department of Human Molecular Genetics and Biochemistry, Sackler School of Medicine, Tel Aviv University, Tel Aviv 69978, Israel; ^2^ Gynecologic Oncology Division, Hillel Yaffe Medical Center, Affiliated with the Technion Institute of Technology, Haifa, Hadera 38100, Israel; ^3^ David B. Kriser Dental Center, Department of Basic Science and Craniofacial Biology, New York University College of Dentistry, New York, NY 10010, USA; ^4^ Endocrine and Diabetes Research Unit, Schneider Children’s Medical Center, Petah Tikva 49292, Israel; ^5^ Yoran Institute for Human Genome Research, Tel Aviv University, Tel Aviv 69978, Israel

**Keywords:** insulin-like growth factor-1 (IGF1), IGF1 receptor, ZYG11A, p53, endometrial cancer

## Abstract

The insulin-like growth factors (IGF) have a key role in the development of gynecological cancers, including endometrial tumors. Uterine serous carcinoma (USC) constitutes a defined histological category among endometrial cancers. Laron syndrome (LS) is a genetic type of dwarfism that results from mutation of the growth hormone receptor (*GHR*) gene, and is the best characterized entity under the spectrum of the congenital IGF1 deficiencies. Epidemiological studies have shown that LS patients are protected from cancer development. Recent genome-wide association studies conducted on LS-derived lymphoblastoid cells led to the identification of a series of metabolic genes whose over-representation in this condition might be linked to cancer protection. Our analyses led to the identification of *ZYG11A*, a potential cell cycle regulator, as a new downstream target for IGF1 action. The aim of the present paper was to investigate the regulation of *ZYG11A* gene expression by IGF1 and insulin in endometrial cancer cell lines and to assess the impact of tumor suppressor p53 on *ZYG11A* expression and biological action. Using USC-derived cell lines expressing a wild type or a mutant p53 gene, we demonstrate that IGF1 inhibited *ZYG11A* mRNA and protein levels in cells containing a wild type *p53*. On the other hand, IGF1 potently stimulated ZYG11A expression in mutant p53-expressing cells. Data presented here links the IGF1 and p53 signaling pathways with ZYG11A action. The clinical implications of the present study in endometrial and other types of cancer must be further investigated.

## INTRODUCTION

The insulin-like growth factors (IGF) constitute a complex network of ligands, cell-surface receptors and binding proteins that, in a highly orchestrated manner, regulate growth and metabolism of multiple organs and tissues throughout life [1, 2]. The biological actions of the ligands (IGF1, IGF2) are mediated by the IGF1 receptor (IGF1R), a transmembrane tyrosine kinase-containing heterotetramer that resembles the insulin receptor (INSR) in structural and functional terms [3, 4]. Most experimental and clinical evidence is consistent with the notion that INSR activation (mainly by insulin) is primarily involved in metabolic types of action whereas IGF1R activation (mainly by IGF1 or IGF2) mediates predominantly growth and differentiative activities [5–7]. The large homology between ligands and receptors within this growth factor family results in a significant degree of cross-talk that leads to a remarkable level of biological complexity.

Epidemiological studies conducted over the past two decades provide firm evidence that high circulating IGF1 levels correlate with an increased cancer risk [8, 9]. This paradigm is particularly true in a number of adult epithelial tumors typically associated with the endocrine system, including breast and prostate cancers [10, 11]. The epidemiological association between IGF1 and cancer incidence is conceptually consistent with the prosurvival and anti-apoptotic roles of IGF1 [12–14]. On the other hand, a putative correlation between low IGF1 dosages and cancer risk has not yet been investigated in a systematic fashion.

Laron syndrome (LS), or primary growth hormone resistance, is a genetic type of dwarfism that results from mutation of the growth hormone receptor (GHR) gene and that is transmitted in an autosomal recessive fashion [15–17]. LS falls within the umbrella of the congenital IGF1 deficiencies and has been thoroughly characterized over the past half century on clinical, endocrine and metabolic grounds [18, 19]. Epidemiological studies conducted on two independent cohorts provide evidence that LS patients are protected from cancer development [20, 21]. To identify genes and pathways that might be differentially represented in LS and that might be associated with cancer protection, we have recently conducted genome-wide association studies on lymphoblastoid cell lines derived from LS patients [22]. Our analyses led to the identification of a series of genes that are either over- or under-represented in LS patients in comparison to healthy controls and that can, potentially, be responsible for cancer evasion in this condition. Among other genes, ZYG11A mRNA levels were more than 3-fold higher in LS-derived than in age-, gender-, and ethnicity-matched control cells.

*ZYG11A* is a member of the *ZYG11* gene family. *ZYG11A* was originally cloned by Pawlak *et al.* and defined as a potential cell cycle regulator [23]. Subsequent studies revealed that the *ZYG11* gene family is involved in cell division during meiosis [24]. Its homologue, ZYG11B, was reported to serve as a substrate recruitment subunit for a cullin-2-based E3 ubiquitin ligase [25, 26]. Dysregulation of the cullin-2-based E3 ubiquitin system is associated with numerous human diseases, including cancer, and was correlated with the prognosis of cancer patients. *ZYG11A* has not been previously linked to the IGF1 signaling pathway. Furthermore, no information is available regarding the potential involvement of tumor suppressor p53 in regulation of *ZYG11A* gene expression and action.

Endometrial cancer is the most common gynecologic malignancy in the Western world [27]. Endometrial tumors are divided into two main groups on the basis of morphological, clinical and molecular parameters [28]. Type I tumors constitute ~80% of the cases, are usually well-differentiated and have a relatively good prognosis. Type II tumors, on the other hand, occur in older women, are more aggressive and have a worse prognosis [29]. Uterine serous carcinomas (USC) constitute the most important histological subtype among Type II tumors. Mutations of the *p53* gene appear early in the course of the disease and are regarded as a paramount genetic factor in the initiation of USC [30]. Overexpression of p53 in endometrial carcinoma is usually correlated with advanced disease and poor prognosis [31]. Epidemiological studies reported a marked increase in the incidence of endometrial cancer over the past few decades, most probably as a result of the spreading obesity epidemic [32]. Sedentary lifestyle, overweight and insulin resistance are considered important risk factors for this type of gynecologic cancers.

In view of the emerging connection between the insulin/IGF system, obesity and diabetes with endometrial cancer etiology and progression, and given our recent identification of the *ZYG11A* gene as a candidate downstream target for IGF1 action, we investigated in this paper the regulation and action of ZYG11A in endometrial cancer cell lines. In addition, we addressed the impact of *p53* mutational status on ZYG11A expression and biological function. Our data suggest that the *ZYG11A* gene constitutes a novel target for IGF1 action in endometrial cancer cells. Regulation of *ZYG11A* expression by IGF1 is dependent on p53 status although abrogation of ZYG11A action leads to inhibition of proliferation and increase in apoptosis in a p53-independent fashion. The biological and clinical implications of the IGF1-ZYG11A connection merits further investigation.

## RESULTS

### Identification of ZYG11A as a potential target for IGF1 action

Recent genome-wide association studies conducted on LS patients identified *ZYG11A* as a highly represented gene in this genetic type of dwarfism [22]. Specifically, ZYG11A mRNA levels were more than 3-fold higher in LS-derived than in control-derived lymphoblastoid cell lines. Genomic results were validated by qRT-PCR (data not shown). These results constitute the conceptual framework upon which the present study is based. Specifically, given the decreased levels of circulating and locally-produced IGF1 in this pathology, we postulated that elevated ZYG11A concentrations in LS might result from relaxation of inhibitory regulation by IGF1.

### Regulation of ZYG11A gene expression by IGF1 in endometrial cancer cell lines

**Figure 1:**
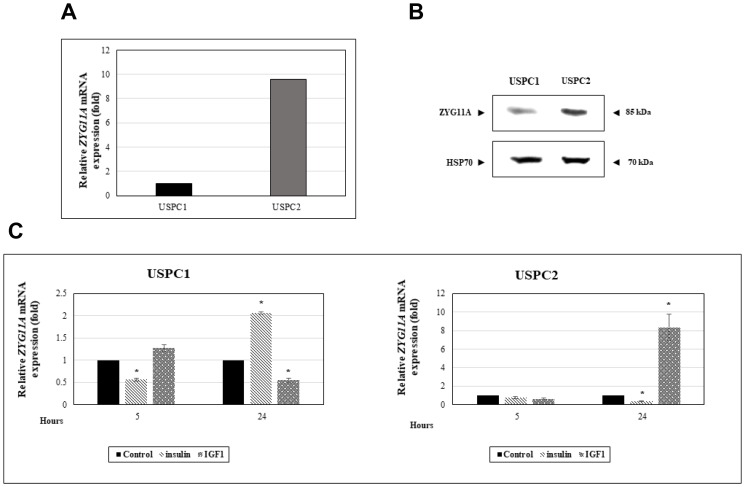
Regulation of *ZYG11A* gene expression by IGF1 in endometrial cancer cells. (**A**) USPC1 (wild type p53-expressing) and USPC2 (mutant p53-expressing) endometrial cancer cell lines were grown to confluence, after which total RNA was prepared and ZYG11A mRNA levels were measured by qRT-PCR. GAPDH mRNA levels served as an internal control. (**B**) USPC1 and USPC2 cells were maintained in serum-free conditions for 24 hr, after which cells were harvested, total protein was prepared, and ZYG11A levels were measured by Western blots. Levels of HSP70 were measured as a loading control. (**C**) USPC1 and USPC2 cells were starved for 24 hr, after which they were treated with 50 ng/ml IGF1 or insulin. At the end of the incubation period, RNA was prepared and ZYG11A mRNA levels were measured as described above. The bars represent the mean ± SEM of three independent experiments, performed in triplicate. ^*^ *p* < 0.01 *versus* respective control.

In view of the epidemiological and clinical correlations between the insulin/IGF1 signaling pathway and endometrial cancer risk and to explore the potential regulation of *ZYG11A* by IGF1, we investigated the expression of this gene in endometrial cancer-derived cell lines. The USPC1 and USPC2 cell lines were derived from USC patients who experienced rapid tumor progression during adjuvant chemotherapy after primary surgical debulking [33]. Mutational analysis of the *p53* gene in the USPC2 cell line revealed a homozygote C to T nucleotide exchange (exon 5: position c.493) that results in the formation of a stop codon at position p.165 [34]. The USPC1 cell line included two polymorphic changes in the *p53* gene sequence (intron 3: c.96+41del16bp; exon 4: c.97-29C > A. To evaluate the impact of *p53* mutational status on *ZYG11A* gene expression, confluent cells were harvested and ZYG11A mRNA levels were measured by qRT-PCR. mRNA levels were normalized to the internal control GAPDH mRNA. Results obtained revealed that basal ZYG11A mRNA levels were 9-fold higher in mutant *p53*-containing USPC2 cells than in wild-type *p53*-expressing USPC1 cells (Figure 1A). These results were corroborated by Western blot analyses showing that, under starving conditions, ZYG11A protein levels were significantly higher in USPC2 than in USPC1 cells (Figure 1B).

To evaluate the effect of insulin or IGF1 on ZYG11A mRNA expression, USPC1 and USPC2 cell lines were serum-starved for 24 hr, after which they were treated with either insulin or IGF1 (50 ng/ml) for an additional 24 hr. qRT-PCR measurements revealed that, in USPC1 cells, IGF1 treatment decreased ZYG11A mRNA levels by 45% whereas insulin increased ZYG11A mRNA expression by 2-fold (Figure 1C). On the other hand, insulin induced a major (65%) reduction in ZYG11A mRNA levels in mutant p53-expressing USPC2 cells whereas IGF1 stimulated gene expression by 8-fold. No major effects on *ZYG11A* gene expression were seen after 5 hr of hormonal treatments (with the exception of a transient insulin-induced decrease in ZYG11A mRNA levels in USPC1 cells).

### Regulation of ZYG11A protein levels by IGF1

**Figure 2:**
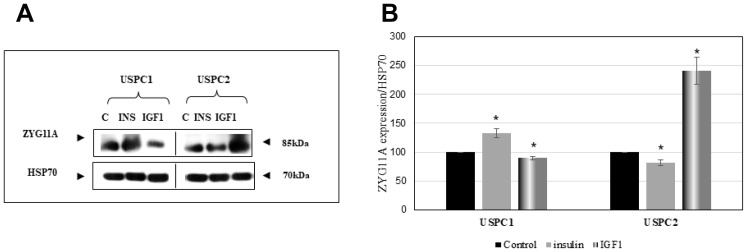
Regulation of ZYG11A protein levels by IGF1. (**A**) Confluent USPC1 and USPC2 cells were treated with insulin (INS) or IGF1 (50 ng/ml) for 24 hr, after which cells were harvested and lysed as described in Materials and Methods. Lysates (130 mg protein) were analyzed by Western blotting for ZYG11A expression. Levels of HSP70 were measured as a loading control. (**B**) Densitometric analysis of ZYG11A regulation by insulin and IGF1. Bars denote the mean ± SEM of three independent experiments. ^*^ *p* < 0.05 *versus* respective control.

To evaluate whether the effects of insulin and IGF1 on ZYG11A mRNA expression described above were accompanied by corresponding changes in protein levels, cells were treated with insulin or IGF1 for 24 hr, after which total protein was isolated and ZYG11A protein levels were measured by Western blotting using a ZYG11A specific antibody. Results obtained indicate that insulin strongly stimulated ZYG11A expression in USPC1, but not USPC2, cells. On the other hand, IGF1 elicited a potent stimulatory effect in USPC2, but not USPC1, cells (Figure 2). Taken together, ZYG11A mRNA changes were in general associated with parallel alterations at the protein level.

### Silencing experiments

**Figure 3:**
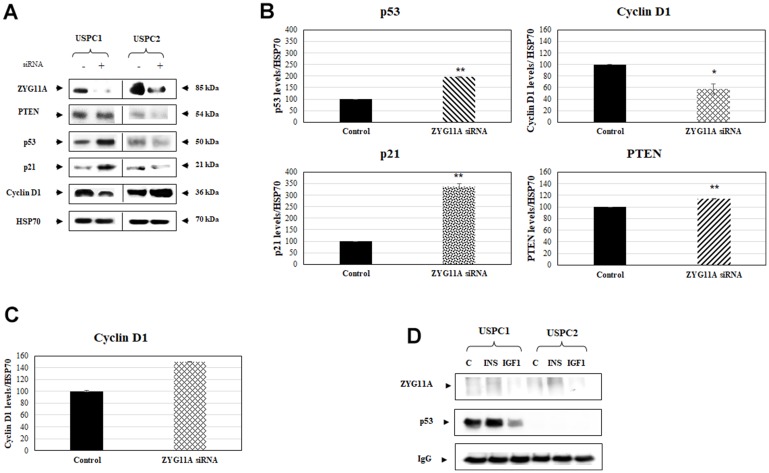
Effect of ZYG11A knockdown on cell cycle regulated proteins. (**A**) USPC1 and USPC2 cell lines were maintained in full medium for 24 hr, after which they were transfected with ZYG11A or non-targeting siRNA for 48 hr. At the end of the incubation period, lysates (130 mg) were analyzed by Western blotting for ZYG11A, pTEN, p53, p21 and cyclin D1 levels. Levels of HSP70 were measured as a loading control. (**B**) Scanning densitometry of p53, cyclin D1, p21 and pTEN expression in ZYG11A-knockdown USPC1 cells. *p<0.05, **p<0.01 *versus* respective control. (**C**) Scanning densitometry of cyclin D1 expression in ZYG11A-knockdown USPC2 cells. (**D**) USPC1 and USPC2 cells were treated with insulin or IGF1 (or left untreated, controls) for 24 hr, after which extracts were prepared. Cell extracts were immunoprecipitated with anti-p53, followed by SDS-PAGE and immunoblotting with anti-ZYG11A or anti-p53. IgG was used as a control for the co-IP experiments.

To examine the effect of ZYG11A knockdown on key cell cycle regulatory proteins and, furthermore, to evaluate the impact of a mutant *p53* gene on ZYG11A activity, USPC1 and USPC2 cells were seeded into 6-cm plates and, after 24 hr, were transfected with a specific siRNA against *ZYG11A*. Optimal siRNA concentrations were determined by extensive calibration experiments (data not shown). Total protein was isolated at the indicated time points and Western blot analyses were performed as described in *Materials and Methods*. Results obtained indicate that p53 and p21 (a downstream target of p53) expressions were largely enhanced in USPC1 cells with a silenced ZYG11A compared to control NT siRNA- transfected cells. On the other hand, cyclin D1 (a positive cell cycle regulator) expression was markedly reduced in ZYG11A-knocked down USPC1 cells. Finally, the expression levels of pTEN were similar in both ZYG11A-silenced and control cells (Figure 3A, 3B).

Given the fact that the *p53* gene is mutated in USPC2 cells (and, therefore, undetectable by Western blots), ZYG11A silencing had no further effect on the expression of p53 and pTEN in this cell line. On the other hand, ZYG11A knockdown led to a small but significant increase in cyclin D1 expression (Figure 3C). Combined, results of silencing experiments highlight a potential functional correlation between ZYG11A and a number of classical cell cycle mediators. Furthermore, the ability of ZYG11A to modulate the expression of both positive and negative cell cycle regulators is determined to a large extent by the mutational status of tumor suppressor p53.

### Analysis of physical interactions between ZYG11A and p53

To gain further insight into the potential mechanism of action of ZYG11A and, moreover, to evaluate a putative physical interaction with p53, co-immunoprecipitation (co-IP) experiments were conducted. For this purpose, USPC1 and USPC2 cells were immunoprecipitated with anti-p53, electrophoresed through 10% SDS-PAGE, and blotted with anti-p53 or anti-ZYG11A. Co-IP experiments revealed no physical association between ZYG11A and p53, suggesting that the functional interplay between both proteins involves, most probably, additional cellular players and/or formation of multimeric protein complexes (Figure 3D).

### Cell proliferation assays

**Figure 4:**
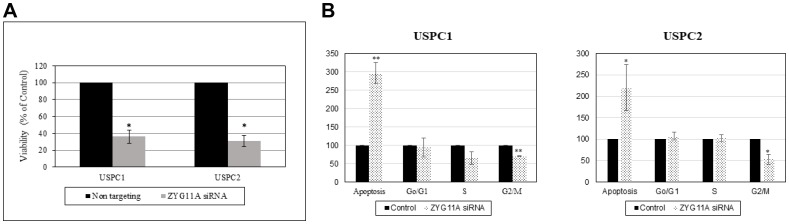
Effect of ZYG11A silencing on cell proliferation and cell cycle distribution. (**A**) USPC1 (2.3 × 10^3^ cells/well) and USPC2 (1.7 × 10^3^ cells/well) cells were plated in 6-well plates in serum-containing medium and, after 24 hr ZYG11A gene expression was knockdown using specific (or NT) siRNA as described in Materials and Methods. Cell proliferation was measured after 48 hr using an XTT kit. Result of a representative experiment repeated three times with similar results are shown. ^*^ *p* < 0.01 versus controls. (**B**) USPC1 and USPC2 cells were seeded in quadruplicate dishes and transfected with ZYG11A (dotted bars) or NT (solid bars) siRNA for 48 hr. Cell cycle distribution was assessed by FACS analysis.

Next, we investigated the effect of ZYG11A expression on endometrial cancer cell proliferation. To this end, the *ZYG11A* gene was silenced as described above, followed by XTT cell proliferation assays. ZYG11A siRNA-transfected USPC1 and USPC2 cells showed major reductions (64% and 70%, respectively, at 48 hr) on proliferation rates compared to control cells (Figure 4A). These results suggest that ZYG11A exhibits proliferative activities both in the presence of a wild type or a mutant *p53* gene.

### Apoptosis assays

To address the effect of ZYG11A on cell cycle progression in endometrial cell lines, ZYG11A was knocked down for 48 hr (USPC1 cells) or 72 hr (USPC2 cells). At the end of the incubation period, cells were washed with PBS, trypsinized, permeabilized with Triton X-100 (4%) and stained with propidium iodide (50 ng/ml). Stained cells were analyzed using a FACSort flow cytometer. Results obtained revealed an almost 3-fold increase in the proportion of apoptotic USPC1 cells following ZYG11A knock down. In addition, silencing led to a reduction of 30% in the portion of cells at the G2/M phase and a 50% reduction in cells at S phase (Figure 4B). In USPC2 cells, ZYG11A-silenced cells exhibited a 2-fold increase in the proportion of apoptotic cells compared to control. In addition, there was a significant reduction of 58% in G1/M phase and a slight increase in S phase. Taken together, these results show that the *ZYG11A* gene affects cell cycle progression in a similar fashion in both wild type- and mutant-p53-expressing cells. Hence, the potentially protooncogenic properties of ZYG11A seem to be independent of p53 status.

### Expression of ZYG11A in breast cancer cell lines

**Figure 5:**
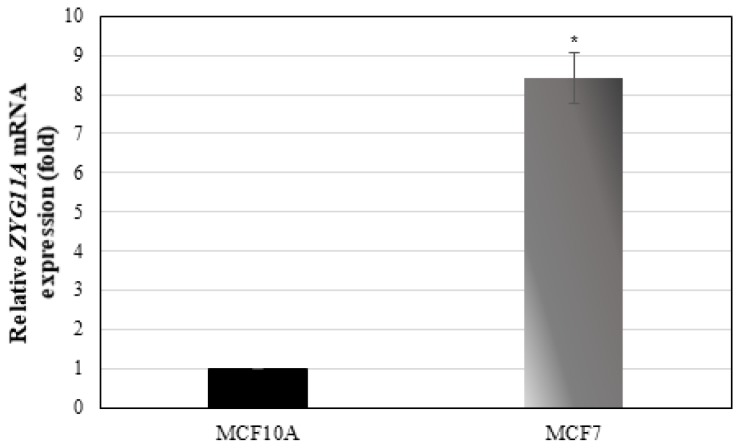
ZYG11A mRNA expression in breast cancer cell lines. Confluent MCF7 and MCF10A cells were harvested and total RNA was prepared. ZYG11A mRNA levels were measured by qRT-PCR as described in Materials and Methods. Bars denote ZYG11A mRNA values normalized to corresponding β-actin levels. Result of a representative experiment repeated three times with similar results are shown. ^*^ *p* < 0.01 versus controls.

To evaluate whether ZYG11A is expressed in additional tumor-derived cell lines and, in particular, to assess a potential correlation between ZYG11A mRNA levels and tumorigenic potential, gene expression was measured in the breast cancer-derived MCF7 and MCF10A cell lines. MCF7 is a highly tumorigenic cell line whereas MCF10A is a benign breast-derived line. qRT-PCR revealed that ZYG11A mRNA levels were 8.7-fold higer in MCF7 than in MCF10A cells (Figure 5). Hence, these results support the notion that ZYG11A expression is likely correlated with increased tumorigenicity.

### Animal studies

**Figure 6:**
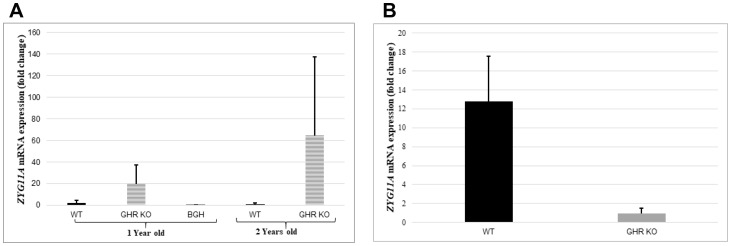
Animal studies. Liver tissue of one- and two-years old GHR-KO, bGH and WT mice, and kidney tissue of one-year old GHR-KO and WT animals was obtained and total RNA was prepared as described in Materials and Methods. Zyg11a mRNA levels were measured by qRT-PCR and normalized to the corresponding β-actin mRNA value. Bars represent mean ± SD of 4–5 animals.

The mRNA expression profile of the *Zyg11a* gene was determined by relative gene expression in the liver and kidney of one- and two-year-old GHR−/−, WT and bGH (overexpressing the GH gene) mice by qRT-PCR. Growth hormone receptor ablation was found to affect levels of Zyg11a mRNA differentially in both liver and kidney tissues. In the case of liver, substantial elevations in levels of Zyg11a mRNA in GHRKO animals at both one and two years of age were measured (10- and 63-fold increases compared to WT animals at the same ages, respectively) (Figure 6A). In contrast, mitigation of *Zyg11a* gene expression (86% decrease) was perceived in one year-old bGH animals in comparison to WT mice. Combined, these results confirm the inverse correlation between the GH-IGF1 axis and ZYG11A expression. In kidney tissues, on the other hand, the *Zyg11a* gene was surprisingly much suppressed in conditions associated with deletion of GHR in comparison with WT animals (Figure 6B). These divergent patterns of expression may reflect the fact that the *Zyg11a* gene undergoes tissue-specific regulation. The dependence of tissue Zyg11a levels on locally-produced IGF1 as well as on additional tissue factors remains to be investigated.

## DISCUSSION

The GH-IGF pathway has been implicated in the etiology of several epithelial malignancies, including breast, colon, prostate and gynecologic cancers [8]. Whereas high circulating IGF1 has been typically correlated with an enhanced risk of developing cancer, independent epidemiological surveys conducted on two cohorts of LS and other congenital IGF1 deficient patients generated evidence that low endocrine IGF1 concentrations may, in fact, be associated with a diminished cancer incidence [20, 21]. Recently conducted genome-wide association studies identified a series of genes and signaling pathways that are either under- or over-represented in LS patients and that might be associated with cancer protection in this pathology [22]. Of relevance, our profiling analyses identified a collection of metabolic genes whose expression was enhanced in LS-derived lymphoblastoid cells and whose mechanism of action might lead to protection from oxidative, genotoxic and other cancer-promoting insults [35].

The present study identified the *ZYG11A* gene as a target for inhibitory regulation by IGF1. Consistent with the reduced IGF1 dosages in LS patients, we provided genomic and qRT-PCR proof that ZYG11A mRNA levels were 3-fold higher in LS-derived lymphoblastoid cells than in healthy controls [22]. A similar trend was seen in livers (but not kidneys) of GHRKO mice (‘Laron mouse’). The *ZYG11A* gene family has not been previously linked to the IGF1 signaling pathway. Our focus on endometrial cancer stems from the fact that this tumor is tightly correlated with obesity and, in particular, with the insulin/IGF1 signaling pathways [31]. Paradoxically, LS patients are protected from this (and other types of) tumor, despite their characteristic obesity. On the basis of data generated by profiling of LS patients, our overall aim was to delineate IGF1-dependent metabolic pathways associated with endometrial cancer protection.

In normal endometrium, cyclic changes in IGF1 expression and signaling play a key role in regulating the transition of the premenopausal endometrium through proliferative, secretory and menstrual cycles [36]. IGF1R expression is significantly higher in endometrial carcinoma than in normal endometrium. However, the association between serum IGF1 levels and endometrial cancer risk, and the diagnostic or prognostic value of this correlation, is still an unsettled issue [31]. In this context, it has been shown that additional circulating or locally-produced factors can positively or negatively impinge upon IGF axis component. Furthermore, the IGF1R emerged in recent years as a promising therapeutic target in oncology, including endometrial tumors [37, 38]. However, there is an urgent need to identify biomarkers that can predict and/or monitor responsiveness to IGF1R-directed therapies.

*p53*, a known tumor suppressor gene involved in carcinogenesis of several tissues including uterine cancer, has been linked to the IGF signaling axis [39, 40]. Specifically, it has been shown that p53 regulates *IGF1R* gene expression in endometrial cancer *via* repression of the *IGF1R* promoter [34]. Pathologic deregulation of *IGF1R* gene expression as a result of tumor-specific, loss-of-function *p53* mutations may lead to increased cell surface IGF1R concentrations, with ensuing enhancement of receptor phosphorylation by endocrine or locally-produced IGF1 or IGF2 [41, 42]. However, no information is available regarding the impact of *p53* mutational status on basal *ZYG11A* gene expression as well as on the ability of IGFs to regulate this gene. Results of qRT-PCR and Western analyses demonstrate that ZYG11A levels were significantly higher in the USPC2 cell line, containing a mutant p53, than in USPC1 cells, expressing a wild type 53. In terms of IGF1/insulin regulation of ZYG11A expression, our analyses show that *ZYG11A* gene expression was downregulated by IGF1 in USPC1 cells while in USPC2 cells an opposite pattern of regulation was observed. If corroborated by additional, larger studies, our results might indicate that the *ZYG11A* gene is expressed at higher levels in more severe types of malignancy, or at advanced stages, which in many cases are associated with p53 mutations. This possibility is supported by results showing higher ZYG11A mRNA levels in malignant breast cancer-derived MCF7 cells than in benign MCF10A cells. Finally, co-IP assays provide no evidence of physical (protein-protein) interaction between ZYG11A and p53. The physical and functional interactions between ZYG11A and p53 must be further dissected.

Our results confirm the hypothesis that ZYG11A is involved in cell cycle regulation as well as in the expression of a series of classical cell cycle regulatory genes. As expected from a putative protooncogene, ZYG11A silencing in USPC1 cells led to augmented levels of tumor suppressors p53 and p21 and reduced levels of the cyclin D1 oncogene. The mechanism of action of ZYG11A, however, requires a functional wild type p53 pathway as indicated by the fact that no effects were seen in mutant p53-containing cells. Of biological relevance, ZYG11A silencing led to a marked decrease in proliferation and increase in apoptotic cells regardless of the mutational status of p53. These results reflect the complexity of the IGF1-ZYG11A-p53 regulatory loop in cellular physiology and suggest that additional, yet to be identified, co-regulators are involved in ZYG11A action. Future studies must address the paradoxical high expression of ZYG11A in LS, a condition associated with cancer protection. These studies must include IGF1 treatments of ‘Laron mice’ and re-evaluation of ZYG11A levels as well as analyses of cell cycle proteins.

While it is difficult to compare results obtained in LS-derived cells (expressing a wild type *p53*) to those generated in endometrial cancer cells, we assume that the potentially pro-oncogenic activities elicited by ZYG11A are abrogated by the low levels of IGF1 that prevail in LS. Additional pathways that might be involved in cancer protection in this pathology include the ubiquitin complex and mitochondrial enzymes, including TXNIP [43]. In agreement with our results, Wang et al have recently identified *ZYG11A* as a potential oncogene in non-small cell lung cancer (NSCLC) [44]. The authors demonstrated that ZYG11A was overexpressed in NSCLC compared to adjacent normal tissues. In addition, increased ZYG11A expression was associated with a poor prognosis. Similarly to our data, ZYG11A knockdown in lung cancer-derived cell lines induced cell cycle arrest and inhibited proliferation. Furthermore, knockdown led to decreased expression of cyclin E1, a member of the cyclin family that is required for the transition from G1 to S phase. Of interest, a recent study has shown that the *ZYG11A* gene had significantly lower methylation rates and higher protein expression in invasive lung adenocarcinoma [46].

In summary, our study has identified the *ZYG11A* gene as a new downstream target for IGF1 action, with potential relevance in endometrial cancer biology. Analyses revealed that the effect of IGF1 on *ZYG11A* gene expression depends on p53 status, thus linking the IGF1 and p53 signaling pathways with ZYG11A action. Future studies will investigate the levels of expression of ZYG11A in primary and metastatic tissues in comparison to normal mon-transformed tissue. Finally, the potential role of ZYG11A as a predictive or prognostic factor in IGF1R-directed targeted therapy must be explored.

## MATERIALS AND METHODS

### Cell cultures

The USPC1 and USPC2 uterine serous papillary carcinoma cell lines were used in this study. USPC cells were grown in RPMI-1640 medium supplemented with 10% fetal bovine serum (FBS), glutamine, and antibiotics. Reagents were purchased from Biological Industries Ltd, Beit-Haemek, Israel. Cultures were maintained under a humidified 5% CO_2_ atmosphere at 37°C. USPC cell lines were provided by Dr. A. Santin (Yale University School of Medicine, New Haven, CT, USA).

### Cell treatments

Cells were serum-starved for 24 hr, after which they were treated with 50 ng/ml of IGF1 (PeproTech Ltd., Rocky Hill, NJ, USA) or insulin (Sigma-Aldrich, St. Louis, MO, USA) for different time periods. All experiments were carried out in triplicates.

### Animal studies

The generation of the GHRKO mouse model was previously described [45]. All mice were in the C57BL/6J (B6) genetic background. Weaned mice were allocated randomly into cages separated according to their sex. Mice were housed 2–5 animals per cage in a facility with 12-hr light:dark cycles and free access to food and water. The analyses were performed in one and two years-old mice. All animal procedures were approved by the Institutional Animal Care and Use Committee of the NYU School of Medicine (Assurance number A3435-01, USDA license No. 465), and conform to the Animal Research: Reporting of In Vivo Experiments (ARRIVE) guidelines (http://www.nc3rs.org.uk/arrive-guidelines). RNA from liver and kidney tissues was extracted with an RNAeasy plus Mini Kit (Qiagen). Approximately 30 mg of tissue sample was homogenized with a Tissue lyser II at 30,000 rpm for 9 min. The homogenized tissues were then applied to gDNA Eliminator Mini Spin Columns and washed extensively with the buffers supplied before eluting RNA in water. Finally, 30 μl DEPC-dH2O was added to the eluted RNA. For cDNA synthesis, 1 ug of RNA was used as per instructions in kit (Superscript III First strand). qRT-PCR analysis was performed using the primers shown in Table 1.

**Table 1:** qRT-PCR primer sequences

	Primer sequence (5’-3’)
Mus musculus Zyg11a	F- GTGGCCTTGAGTCATTTCACT
	R- CCAGGTTCGGTAACTGAGAAAC
Mus musculus 18S ribosomal RNA (Rn18s)	F- GCCGCTAGAGGTGAAATTCTT
	R- CGTCTTCGAACCTCCGACT
Mus musculus β Actin	F- AGATGACCCAGATCATGTTTGAG
	R- TGGTACGACCAGAGGCATACA

### Quantitative real time-polymerase chain reaction (qRT-PCR)

Total RNA from USPC1 and USPC2 cell lines was prepared using the Trizol reagent (ThermoFisher Scientific, Waltham, MA, USA). Two-hundred ng of total RNA was reverse transcribed using the Superscript First-Strand Synthesis system for cDNA synthesis by PCR (ThermoFisher Scientific). qRT-PCR was performed using Faststart Universal SYBR® Green Mix (Sigma-Aldrich). For control purposes, levels of β-actin and GAPDH mRNA were measured. The number of PCR cycles to reach the fluorescence threshold is the cycle threshold (Ct). Each cDNA sample was tested in triplicate and mean Ct values are reported. For each reaction, a “no template” sample was included as a negative control. Fold differences were calculated using the 2ΔΔCt method [46]. Primers are shown in Table 2.

**Table 2:** qRT-PCR primer sequences

	Primer sequence (5′–3′)
Homo sapiens Zyg11a	F- CGGAGCATTGGAGTTTCCCT
	R- CTGTCAGTCAGCTTGCCTTG
Homo sapiens β Actin	F- CCTGGCACCCAGCACAAT
	R- GGGCCGGACTCGTCATACT
Homo sapiens GAPDH	F- GCGCACCGTCAAGGCTGAGAAC
	R- AATGGTGGTGAAGACGCCAGT

### Western blot analyses

Confluent cells were washed with ice-cold phosphate-buffered saline (PBS) containing 5 mM EDTA and centrifuged at 1100 rpm. After discarding the supernatant, lysis buffer was added and the cells were incubated on ice for 10 min. Cells were then centrifuged at 13000 rpm for 10 min. Protein concentration was determined by the Bradford method. Samples were electrophoresed through 10% SDS-PAGE, followed by blotting of the proteins onto nitrocellulose membranes. After blocking with 5% skim milk, the blots were incubated overnight with antibodies against ZYG11A (Abcam plc, Cambridge, UK) and heat shock cognate protein 70 (#Hsp73, Sigma-Aldrich). In addition, antibodies against pTEN (#9559), cyclin D1 (#2926) and p21 Waf1/Cip1 (#2947) were obtained from Cell Signaling Technology (Danvers, MA, USA). Anti-p53 (mixture of DO-1 and Pab 1801) was purchased from Santa Cruz Biotechnology (Santa Cruz, CA, USA). After incubation, the blots were washed and incubated with the appropriate horseradish peroxidase-conjugated secondary antibody. Proteins were detected using the enhanced chemiluminescence reaction Westar Supernova (Cyanagen, Bologna, Italy]. HSP70 was used as a loading control.

### Co-immunoprecipitation (Co-IP) assays

Total lysates (500 μg) were diluted 1:2 with IP dilution buffer [1% Triton X-100, 150 mM NaCl, 20 mM Tris buffer (pH 7.5) containing proteases and phosphatases inhibitors], and immunoprecipitated by incubating overnight at 4°C with anti-p53 (#DO-1; Santa Cruz Biotechnology). Protein A/G-agarose beads (#SC-20003; Santa Cruz Biotechnology) were added to the samples and incubated for 2 hr. Samples were then washed with PBS, mixed with sample buffer, boiled for 10 min at 95°C, and electrophoresed through 10% SDS-PAGE. Finally, membranes were blotted with anti-p53 or anti-ZYG11A, as described above.

### Silencing experiments

For small interference RNA (siRNA) knockdown of ZYG11A, siRNA against the human ZYG11A and non-targeting (NT) siRNA were purchased from Dharmacon Research Inc (Lafayette, CO, USA) (Table 3). USPC1 and USPC2 cells were transfected using INTERFERin (Polyplus Transfection Inc, Illkirch, France). Briefly, cells were seeded into 6-well plates the day before transfection, and doses of 5, 7.5 or 10 nM of siRNA were used for each transfection. ZYG11A knockdown was tested after 48, 72 and 96 hr by immunoblotting.

**Table 3:** Target sequences for human ZYG11A (A) and non-targeting (NT, B) siRNA

A.
AAUCAAGGAUUGCAAAUCU
CAGCAUUGGUGACCUAUAG
UAUCAUAGCCCACCUGACA
GAAUGAGGAAUCACCCAUU

B.
UAAGGCUAUGAAGAGAUAC
AUGUAUUGGCCUGUAUUAG
AUGAACGUGAAUUGCUCAA
UGGUUUACAUGUCGACUAA

### Proliferation assays

Cell proliferation was monitored using an XTT cell proliferation kit (Biological Industries Ltd) according to manufacturer’s instructions. Twenty-four hr post-siRNA transfection, cells were seeded in 96-well plates and twenty-four hr later XTT reagent was added. One to two hours after addition of the XTT reagent, sample absorbance was measured with a spectrophotometer at a wavelength of 530 nm and reference wavelength of 630 nm. on a UVmax Kinetic Microplate Reader (Molecular Devices Inc, Sunnyvale, CA, USA).

### Cell cycle assays

Twenty-four hr prior to siRNA addition, cells were split into 6-well plates (1x10^5^ cells/well). After 24 hr, transfection with siRNA or NT was conducted. After an additional 24, 48 and 72 hr incubation (according to each cell lines’ optimal siRNA concentration and time point), cells were washed three times with PBS, trypsinized, permeabilized with Triton X-100 (4%) and stained with propidium iodide. Stained cells were analyzed using a FACSort flow cytometer (Becton Dickinson, Franklin Lakes, NJ, USA).

### Statistical analyses

The statistical significance of differences between groups was assessed by Student’s t-test (two samples, equal variance). *p* values < 0.05 were considered statistically significant.
